# Site‐specific tolerance tables and indexing device to improve patient setup reproducibility

**DOI:** 10.1120/jacmp.v16i3.5097

**Published:** 2015-05-08

**Authors:** Kiernan T. McCullough, Joshua A. James, Ashley J. Cetnar, Mark A. McCullough, Brian Wang

**Affiliations:** ^1^ Department of Radiation Oncology University of Louisville School of Medicine Louisville KY; ^2^ Colorado Associates in Medical Physics Colorado Springs CO USA

**Keywords:** indexing, couch tolerances, record and verify, patient setup

## Abstract

While the implementation of tools such as image‐guidance and immobilization devices have helped to prevent geometric misses in radiation therapy, many treatments remain prone to error if these items are not available, not utilized for every fraction, or are misused. The purpose of this project is to design a set of site‐specific treatment tolerance tables to be applied to the treatment couch for use in a record and verify (R&V) system that will insure accurate patient setup with minimal workflow interruption. This project also called for the construction of a simple indexing device to help insure reproducible patient setup for patients that could not be indexed with existing equipment. The tolerance tables were created by retrospective analysis on a total of 66 patients and 1,308 treatments, separating them into five categories based on disease site: lung, head and neck (H&N), breast, pelvis, and abdomen. Couch parameter tolerance tables were designed to encompass 95% of treatments, and were generated by calculating the standard deviation of couch vertical, longitudinal, and lateral values using the first day of treatment as a baseline. We also investigated an alternative method for generating the couch tolerances by updating the baseline values when patient position was verified with image guidance. This was done in order to adapt the tolerances to any gradual changes in patient setup that would not correspond with a mistreatment. The tolerance tables and customizable indexing device were then implemented for a trial period in order to determine the feasibility of the system. During this trial period we collected data from 1,054 fractions from 65 patients. We then analyzed the number of treatments that would have been out of tolerance, as well as whether or not the tolerances or setup techniques should be adjusted. When the couch baseline values were updated with every imaging fraction, the average rate of tolerance violations was 10% for the lung, H&N, abdomen, and pelvis treatments. Using the indexing device, tolerances for patients with pelvic disease decreased (e.g., from 5.3 cm to 4.3 cm longitudinally). Unfortunately, the results from breast patients were highly variable due to the complexity of the setup technique, making the couch an inadequate surrogate for measuring setup accuracy. In summary, we have developed a method to turn the treatment couch parameters within the R&V system into a useful alert tool, which can be implemented at other institutions, in order to identify potential errors in patient setup.

PACS numbers: 87.53Kn, 87.55.kh, 87.55.ne, 87.55.km, 87.55K‐, 87.55.Qr

## I. INTRODUCTION

The reproducibility and accuracy of patient setup has become an increasingly important area of focus in radiotherapy as higher dose is delivered on every fraction of treatment for emerging hypofraction and dose escalation schemes. Image‐guidance and immobilization devices are commonly used to reduce setup errors in these high‐dose techniques, but these methods can be costly and time‐consuming for conventionally fractionated treatment. Without these safety measures, conventional treatments are more susceptible to treatment incidents or near‐misses. Errors could also arise from many additional factors, such as confusion with multiple tumor sites, errors in imaging alignment, loss of laser alignment marks, or other operator related mistakes. Ford et al.^(1)^ summarized quality control checks to catch incidents or near‐misses from two institutions. In their study, the sensitivities of 15 different commonly used quality control measures were analyzed. The most optimum combination of these measures was able to detect 97% of potential treatment errors when implemented correctly. A couch tolerance system would serve to decrease the frequency of these errors even further, by alerting therapists to potential oversights and act as a redundant safety measure to quality control procedures already in place.

A group of investigators at the University of Florida has developed an automatic patient safety system to prevent gross setup errors.^(2)^ Their system consists of a pair of charge‐coupled device cameras and infrared reflective markers attached on patients or immobilization devices. Their in‐house program compares the locations of infrared markers at the treatment position to those from the computed tomography (CT) simulation position. The developed system has caught one potential gross setup error that would have gone undetected and resulted in treating the wrong site.

Several commercial systems can also be used for patient setup and monitoring using optical guidance technologies.^3^, ^4^, ^5^ The limitation of such commercial and customized systems is their limited availability to other radiation therapy centers. On the other hand, almost all centers are currently equipped with an electronic record and verify (R&V) system which can be used to monitor actual treatment couch position versus expected position.^(6)^ The goal of this study is to design and implement a set of site‐specific treatment couch tolerance tables within an existing R&V system in order to prevent gross setup errors. In fact, as stated in the paper, gross errors caught by the customized system at the University of Florida would have required an override of the treatment couch position in the R&V system. Therefore, our proposed method will fulfill the purpose of preventing gross setup errors, and it can be adopted by other centers using their existing R&V system. We also developed a simple indexing device to improve the reproducibility of patient setup positions with minimal workflow interruption, which can also be replicated at other centers.

Some groups have previously explored the option of using couch position tolerance tables to detect mistakes in patient setup. Patton et al.^(7)^ reviewed all the treatment errors from one institution and found inappropriate tolerance tables to be one type of error related to the R&V system. Hadley et al.^(8)^ compared the sensitivity and specificity between two baselines to calculate tolerance: a first day position and a cumulative average over previous fractions. They found that it was better to use the average as a baseline instead of the first day's couch parameters. They also concluded that different treatment sites require different tolerance tables. One weakness of their study is that the calculated tolerances were not implemented into clinical practice for validation. In this study, we implemented the tolerance tables and report the effectiveness of this method. In addition, we also present a different method to calculate tolerances by adjusting the baseline after every fraction with image guidance.

## II. MATERIALS AND METHODS

### A. Patient selection and generation of tolerance tables

Initially, a committee of physicists, dosimetrists, and therapists from the University of Louisville identified treatment groups based on disease site and method of immobilization. This effectively separated the patient population into three categories: stereotactic body radiation therapy (SBRT) with vacuum‐suctioned bag immobilization, conventional radiation therapy, and clinical setup treatments with highly variable positions. In our clinical workflow, SBRT patients receive cone‐beam computed tomography (CBCT) for alignment that is verified by at least one therapist, physicist, and physician before any treatment fraction is delivered. This patient group already has several layers of safety checks in place and is closely monitored by many members of the staff. Therefore, it was not deemed necessary to develop an additional safety measure using the R&V system. The SBRT group also receives fewer fractions and makes up a low percentage of our total treatments. For the clinical setup group with unique electron or extremity treatments, patients have such a varied setup that the treatment couch parameters are not a very meaningful representation of treatment accuracy. Again, this group is a minority of our patient population. Therefore, the focus of this project was geared towards those patients receiving conventional fractionated therapy since they are both at risk for a potential treatment incident and the treatment couch parameters could be used to effectively guide treatment decision‐making. This group also represents the majority of our patients and those with a high number of fractions, making up most of the clinical workload.

The patients undergoing conventional therapy were parsed into five categories: lung, head and neck (H&N), breast, pelvis, and abdomen. A retrospective analysis of completed patients was performed on a total of 66 patients and 1,308 treatment fractions from December 2008 to March 2013, using ARIA Chart QA version 10.0 (Varian Oncology Systems, Palo Alto, CA). The couch parameters from the first treatment were used as a baseline and the deviation in setup during subsequent fractions was calculated. Each selected patient was treated using one of the indexing and immobilization devices shown in Table 1. Because there were not sufficient indexed data for the abdominal and pelvis sites, these sites were combined into one category and include pelvis patients with and without immobilization. We also took into consideration that, without a set of enforced tolerance tables, the parameters may differ systematically (e.g., using different notches for the indexing bar on the treatment couch). After data collection, the absolute deviations from the initial treatment position were calculated for the vertical, longitudinal, and lateral couch parameters.

**Table 1:** Disease sites and corresponding indexing devices for initial data collection.

*Site*	*Devices Used*
Lung	Standard Wing Board (CIVCO Medical Solutions, Coralville, IA)
H&N	Type‐S Head‐Only perforated mask (CIVCO Medical Solutions, Coralville, IA) Type‐S IMRT Reinforced Style 27 mask (CIVCO Medical Solutions, Coralville, IA)
Breast	C‐Qual Breastboard (CIVCO Medical Solutions, Coralville, IA) Standard Wing Board
Pelvis Abdomen	Vac‐Lok immobilization bag (Elekta Medical Intelligence, Atlanta, GA) Standard Wing Board

**Figure 1:**
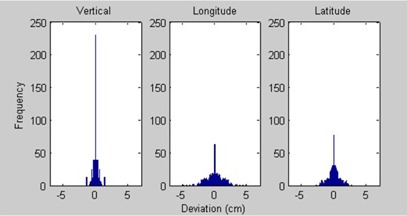
Histogram of data collected for lung disease sites.

Every patient was imaged on the first day of treatment, using either a pair of kV images, CBCT, port films, or a combination of these techniques, and was aligned to bony or soft tissue structures from the treatment planning CT images. In order to simplify the asymmetry of the collected data, we assumed that the direction (positive or negative) of the deviations to be random and insignificant for all three couch parameters. In the analysis, each data point was mirrored about the baseline such that the average treatment position over all fractions is equal to the baseline value. The motivation for this technique was to mimic a normal distribution of values, as shown in the histogram of lung data in Fig. 1. The standard deviations of all three couch parameters were then calculated for all fractions from all patients for a given site. Tolerances were calculated to be 2 standard deviations from the mean, so that the couch parameter deviations fell within the tolerance values for 95% of all treatments, similar to the method used by Hadley et al.^(8)^ This value was chosen in order to achieve a balance between establishing tolerances that were tight enough to prevent gross errors in setup, while avoiding unnecessary tolerance overrides.

### B. Customizable indexing device

**Figure 2:**
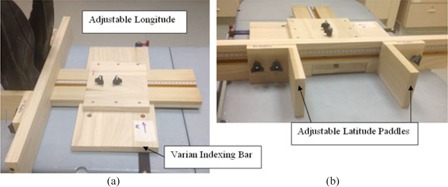
Customized indexing device attached to treatment couch with indexing bar from (a) side view with feet in indexed position, and (b) front view.

There was no effective indexing means with our current equipment for some pelvis patients. Due to the inferior location of the disease site, the couch could not extend far enough to align the patient to isocenter if a wing board or headrest was used. The current setup technique at our institution is the use of a pillow and straight‐leg sponge for the patient's legs, neither of which has indexing capabilities. In order to address this issue, a new customizable indexing device was created with the goal of accessing the same location on the treatment couch repeatedly. This device acts as a reference point for patient setup as well as an accessory to other patient immobilization/indexing devices. As shown in Fig. 2, the device attaches to a standard Varian indexing bar and latches into notches that are spaced every 14 cm. The longitude is indexed by an adjustable plank that can be secured, giving a location to abut the patient's feet or other devices to. It also has two paddles that slide on a track which is implanted in the longitudinal plank, acting as bookends to give a reference for latitude. All adjustable parts of the indexing device move along a measuring tape such that the position of each part can be easily recorded for patient‐specific settings.

### C. Tolerance table implementation and override analysis

A trial period was initiated on one of the accelerators with a variety of treatment sites. All patients were able to be indexed with existing equipment, except for patients with pelvis disease that previously lacked immobilization. For these patients, as shown in Fig. 2(a), our customizable indexing device was used to straighten the legs into the treatment position, where a pillow and additional sponges were arranged as needed. During the trial period, particular attention was paid to patient setup and indexing concerns, with the intention of evaluating and perhaps redesigning both the tolerance tables and the indexing device.

During this period, parameters for patients under treatment on the chosen accelerator were collected and the data were stored in a program written in MATLAB R2013a (MathWorks, Natick, MA) which organized patient identity, site, vertical, longitudinal, and lateral couch values for every treatment. The program then grouped all patients by treatment site and calculated the difference in couch parameters for each fraction versus their baseline. It then calculated the average absolute difference, standard deviation, and couch tolerance values in the same manner as the initial study without the indexing device. The program also displayed every fraction that would have exceeded the predetermined tolerances. In the event that a treatment did exceed the tolerance values, the therapist administering the treatment would verify that the patient setup was correct and, if no adjustments were needed, override the couch parameter and document the reason for the tolerance violation under a physicist's supervision.

Lastly, statistical analysis was performed in order to determine if the methods used to measure the difference in patient setup were consistent between the initial and trial periods. This was done by using the F‐Test and t‐Test of the Analysis ToolPak in Microsoft Excel (Microsoft, Redmond, WA). The “F‐Test Two‐Sample for Variances” was used to determine the significance of the difference in the group variances. The results of the F‐Test then dictated whether the group variances were assumed to be equal or unequal when using the t‐Test tool. A two‐sample Student's *t*‐test was then performed to determine whether the mean errors in patient setup were meaningfully different between the two study groups.

## III. RESULTS

### A. Tolerance tables

Table 2 shows the results from the initial data collection for 66 patients and 1,308 treatment fractions. The tolerances in Table 2 agree with previously published data concerning the uncertainty of patient setup. For example, a study presented by Rosenthal et al.^(9)^ regarding the uncertainty in setup of H&N patients showed a median value of 0.7 cm total setup uncertainty, which is comparable to a 0.9 cm maximum tolerance for H&N patients using our system. We also compared our results with those from Hadley et al.^(8)^ for four anatomical sites. As shown in Table 3, the standard deviations agreed well for most treatment sites, despite the different immobilization and indexing devices used in the two studies. However, the standard deviations for breasts are larger in the longitudinal and lateral directions in our study. We reviewed the setup photos for these patients and found that the difficulty in reproducing their position was due to larger, pendulous, more mobile breast tissue.

**Table 2:** Initial patient treatment statistics of indexed patients.

	*Treatment*		*Vertical (cm)*			*Longitude (cm)*			*Latitude (cm)*		
*Site*	*Pt*	*Tx*	*Avg*	*SD*	*Tol*	*Avg*	*SD*	*Tol*	*Avg*	*SD*	*Tol*
Lung	20	317	0.3	0.5	0.9	1.2	1.6	3.2	0.7	1.1	2.2
H&N	30	656	0.1	0.2	0.5	0.3	0.4	0.9	0.2	0.5	0.9
Breast	8	155	0.5	0.6	1.3	1.0	1.3	2.7	1.5	2.0	4.0
Pelvis Abdomen	8	180	0.3	0.5	0.9	1.6	1.9	3.8	1.3	1.7	3.5
Total	66	1308									

Pt=number of patient;Tx=number of treatment;Avg=Average;SD=standard deviation;Tol=tolerance

**Table 3:** Comparison of standard deviations in this study versus Hadley et al.^(8)^

	$\sigma_{vertical}(cm)$		$\sigma_{longitude}(cm)$		$\sigma_{latitude}(cm)$	
*Site*	*Hadley*	*UofL*	*Hadley*	*UofL*	*Hadley*	*UofL*
Lung	0.3	0.5	1.2	1.6	1.3	1.1
H&N	0.3	0.2	0.4	0.4	0.4	0.5
Breast	1.0	0.6	0.9	1.3	1.3	2.0
Pelvis	0.4	0.5	3.1	1.9	2.0	1.7

### B. Tolerance tables with baseline adjusted after image guidance

Patient position in an indexing device relative to the table can change dramatically as a result of changes in patient weight or their level of anxiety as they become more accustomed to the treatment process. These changes over the course of treatment could lead to significant differences in couch values that are not related to setup error. In order to account for this, we investigated an alternative option for data analysis, where the baseline values were adjusted every time the setup was verified using image guidance. Table 4 shows the tolerance results from the same dataset as Table 2, except that the baselines were adjusted after every fraction with image guidance. When comparing 2, 4, the tolerance values decreased or stayed the same for all sites, except for the pelvis/abdomen group, when resetting the baseline with image guidance versus using the first fraction as a baseline. The decrease in tolerance values is reasonable because the relationship between the verified treatment isocenter and the patient's indexed position is updated every imaging fraction, reducing the number of false alarms generated by gradual changes in patient setup which are not indicative of an error in treatment. The increased tolerance values for the pelvis/abdomen site may be caused by the relatively small patient number and large setup variations for these groups.

**Table 4:** Initial patient treatment statistics of indexed patients with baseline adjustment after image guidance.

	*Treatment*		*Vertical (cm)*			*Longitude (cm)*			*Latitude (cm)*		
*Site*	*Pt*	*Tx*	*Avg*	*SD*	*Tol*	*Avg*	*SD*	*Tol*	*Avg*	*SD*	*Tol*
Lung	20	317	0.2	0.3	0.6	1.0	1.6	3.2	0.7	1.1	2.1
H&N	30	656	0.1	0.2	0.4	0.2	0.4	0.8	0.2	0.5	0.9
Breast	8	155	0.4	0.5	1.0	0.9	1.3	2.6	1.3	1.8	3.6
Pelvis Abdomen	8	180	0.3	0.4	0.8	2.1	2.7	5.3	1.6	2.1	4.2
Total	66	1308									

Pt=number of patient;Tx=number of treatment;Avg=Average;SD=standard deviation;Tol=tolerance

### C. Tolerance tables between initial and trial period

In order to assess our method for generating the tolerance tables, we compared the tolerances generated from the initial dataset and those from the patients treated during the trial period where indexing was actively being practiced. The trial period study included data from 65 patients and 1,054 fractions. All patients were treated using the same immobilization/indexing technique as the initial study shown in Table 1, except that pelvis patients that would not have been previously indexed were indexed using the customizable indexing device.

Table 5 shows the initial patient study group represents our trial study well in terms of tolerance values. When the baseline values were adjusted using image guidance, each newly generated tolerance was less than or equal to those calculated previously with the exception of the breast cases. Similarly, there was good agreement between tolerances when the baseline was established using the parameters from the first fraction for the lung and H&N sites. However, the breast and pelvis tolerances, as well as the abdominal vertical and longitudinal tolerances, increased from the initial to the trial periods. These discrepancies for the pelvis and abdominal tolerances are not unexpected since there was a change in indexing technique, where the initial group included Vac‐Lok immobilization, while the customizable indexing device was implemented for the pelvis patients during the trial period.

**Table 5:** Comparison of tolerances generated from initial survey and trial period.

	*Baseline Adjusted Using Image Guidance*						*Baseline Set From First Fraction*					
	*Vertical (cm)*		*Long (cm)*		*Lat (cm)*		*Vertical (cm)*		*Long (cm)*		*Lat (cm)*	
*Site*	*Initial*	*Trial*	*Initial*	*Trial*	*Initial*	*Trial*	*Initial*	*Trial*	*Initial*	*Trial*	*Initial*	*Trial*
Lung	0.6	0.4	3.2	2.5	2.1	1.8	0.9	0.6	3.2	3.1	2.2	1.9
H&N	0.4	0.2	0.8	0.5	0.9	0.4	0.5	0.2	0.9	0.5	0.9	0.3
Breast	1.0	1.2	2.6	2.8	3.6	5.2	1.3	1.3	2.7	3.0	4.0	5.7
Pelvis	0.8	0.6	5.3	4.3	4.2	3.7	0.9	1.3	3.8	6.3	3.5	4.5
Abdomen	0.8	0.7	5.3	3.3	4.2	2.3	0.9	1.1	3.8	4.2	3.5	2.5

We also used a *t*‐test in order to affirm that the results from the initial and trial groups were in fact comparable. This was accomplished by first using an *f*‐test to determine the significance of the difference in variance between the two groups so that the appropriate *t*‐test could be selected. If the *f*‐value for the group is larger than the threshold value, then the differences in the variances were considered to be significant using an alpha criterion of 0.05.^(10)^ Table 6 catalogs the results from the *f*‐test for each couch parameter, which was used to determine which type of *t*‐test would be used for that particular value — *t*‐test with equal variance or unequal variance. For the *t*‐tests, we used a two‐tailed analysis with an alpha threshold of 0.05 to determine whether the difference of the means of the patient setup error was statistically significant between the initial and trial groups. Table 7 shows the results from the *t*‐tests; a *t*‐value that is greater in magnitude than the threshold value (as noted in the table) is indicative that the means of the two groups are significantly different.^(11)^ When using image guidance to reestablish baselines, none of the anatomical groups showed a meaningful difference between the initial and trial datasets, with the exception of the breast vertical couch parameter. This contrasts dramatically with the results where the baseline is set from the first fraction, where every anatomical group showed a significant difference between the initial and trial datasets.

**Table 6:** F‐values comparing initial and trial groups' error in setup.

	*Baseline Adjusted Using Image Guidance*				*Baseline Set From First Fraction*			
*Site*	$f_{\mathrm{vertical}}$	$f_{\mathrm{long}}$	$f_{\mathrm{lat}}$	$f_{\mathrm{threshold}}$	$f_{\mathrm{vertical}}$	$f_{\mathrm{long}}$	$f_{\mathrm{lat}}$	$f_{\mathrm{threshold}}$
Lung	1.90	1.66	1.43	1.23	2.22	1.29	1.38	1.23
H&N	6.79	2.35	5.90	1.19	4.74	3.26	7.73	1.19
Breast	1.26	1.12	2.07	1.27	1.12	1.45	2.00	1.27
Pelvis	1.76	1.56	1.29	1.30	2.24	2.62	2.00	1.29
Abdomen	1.28	2.56	3.41	1.29	1.60	1.04	1.57	1.29

**Table 7:** Results of *t*‐test comparing the means of setup error in initial and trial groups.

	*Baseline Adjusted Using Image Guidance*				*Baseline Set From First Fraction*			
*Site*	$t_{\mathrm{vertical}}$	$t_{\mathrm{long}}$	$t_{\mathrm{lat}}$	$t_{\mathrm{threshold}}$	$t_{\mathrm{vertical}}$	$t_{\mathrm{long}}$	$t_{\mathrm{lat}}$	$t_{\mathrm{threshold}}$
Lung	0.72	0.68	−1.04	1.96	4.10^a^	4.13^a^	−2.74^a^	1.96
H&N	−0.18	−0.02	−0.26	1.96	−3.12^a^	3.03^a^	4.56^a^	1.96
Breast	−3.47^a^	0.58	0.81	1.97	−9.72^a^	4.78^a^	0.81	1.97
Pelvis	0.02	0.93	−0.55	1.97	1.87	3.01^a^	−3.67^a^	1.97
Abdomen	−0.06	1.01	0.22	1.97	0.37	−3.40^a^	−6.02^a^	1.97

^a^ Values are above *t*‐test threshold indicating a significant difference in means between initial and trial groups.

### D. Tolerance tables between two baseline methods

The results presented in 5, 6, 7 paint a telling picture regarding the effectiveness of our methods, based on both anatomical site and implementation of the system. When regularly updating the patient's baseline parameters using image guidance, the range of the patient's setup is predictable for most cases and the indexing system can act as a reliable surrogate. If the patient's baseline parameters are never updated, then changes in patient setup can have a dramatic impact on the effectiveness of the system and its ability to apply to a general population. The difference in the number of tolerance violations between the two methods of establishing baseline values in Table 8 clearly demonstrates the benefit of updating the baseline values as treatment progresses. Table 8 shows the number of tolerance violations did not differ significantly for the lung and H&N disease sites; however, there would have been a significant increase in the number of overrides for the abdominal and pelvis patients when the baselines were not updated. For the abdominal patients with a constant baseline value, only two patients account for 36 of the 43 violations (84%). In both of these cases, the patient's setup on the first day of treatment, although verified using image guidance, was not representative of future fractions that were also verified using the same method. These results reflect the complexity of the relationship between a patient's skin marks, internal anatomy, and their relative location to an indexing device/treatment couch, and emphasize how important it is to have baseline values that adapt to changes in patient setup.

**Table 8:** Comparison of table tolerance violations during trial period with reference baseline set after each imaging fraction vs. constant after first fraction.

	*Treatment*		*Image Guidance*		*First Fraction*	
*Site*	*Patients*	*Fractions*	#	*%*	#	*%*
Lung	13	214	15	7.0	16	7.5
H&N	15	274	8	2.9	0	0.0
Breast	18	254	66	26.0	62	24.4
Pelvis	10	149	12	8.1	52	34.9
Abdomen	9	163	8	4.9	43	26.4
Total	65	1054	109	10.3	173	16.4

#=number of tolerance violation; %=percent of fractions with a tolerance violation

Updating the baseline values based on imaging reduces the number of tolerance violations to a reasonable frequency for most sites, but remains unsuccessful in characterizing patients undergoing treatment for breast disease. For instance, three patients who received a combined 56 fractions accounted for 24 tolerance violations, which is an average tolerance violation rate of 43%. This contrasts with two other breast patients in the same dataset who together only accounted for two violations in 38 fractions (5%). The setup photos from these patients reveal the difficulty in reproducing the position of larger patients with larger, pendulous, more mobile breast tissue. This is compounded by the fact that a breastboard setup has many more degrees of freedom which could affect how accurate a surrogate the couch table is for patient alignment evaluation. Unfortunately, it seems that, while these tolerances are useful for guiding the setup of smaller patients with fewer reproducibility issues, they may not add significant benefit to those with a naturally more variable setup.

Evaluating the results from each treatment site, we were not able to accomplish our goal of creating tolerance table values that encompassed 95% of treatment fractions; however, we were able to develop a system that did not require an override for an average of 90.0% of treatments for lung, H&N, pelvis, and abdominal sites. While this did not satisfy our initial goal, we believe that a 10% override frequency, in conjunction with the magnitude of our tolerances, will add a significant level of safety without adding a burden to clinical workflow. In the future, the tolerance table values could be updated based on new patient data and changes in patient setup technique to try to meet our initial goal.

## IV. DISCUSSION

While the couch tolerance tables in the R&V safety system may be limited to identifying severe geometric misses, we have seen its utility during the program's design. By providing the therapist team with meaningful tolerance values, they have responded positively by taking more precaution when aligning patients, paying closer attention to trends in patient interfraction setup difference, and noticing potential issues in patient setup reproducibility. There have even been instances where this system could have prevented mistreatments had it been fully implemented. In one recent incident, an H&N patient received lateral kV imaging for alignment, was adjusted accordingly, and then treated, only to discover that the patient was positioned several centimeters off in the lateral direction. The magnification difference between the verification image and the DRR was not great enough to raise an alarm. If the tolerance table system had been implemented, the therapists would have been alerted and an additional check could have prevented the incident. Of course, an additional AP image would also be able to reveal the misalignment, but on the treatment days without image guidance, the tolerance table would have alerted the therapists about the potential incident.

The indexing system offers many clear benefits as an additional layer of security to the radiation treatment process, particularly by identifying instances of large isocentric misses, while additionally helping to encourage a culture of precision and awareness among the therapists. However, there are certainly considerations that should be kept in mind when trying to implement this in one's department. Most importantly, the therapists and physics staff must be willing to put in the necessary effort to identify groups of patients and indexing strategies that will make the tolerances meaningful. The effectiveness of the tolerances is entirely reliant on the ability of the therapists to achieve precision when setting up the patient, which may be difficult for a variety of factors. In our instance, we had to devise an indexing device to account for unreliable setup in pelvis patients. Other departments may choose to change the way the patients are set up, give looser tolerances, or exclude this group entirely.

Another potential weakness in our study is the small patient population for some sites in the initial tolerance generation. Both the breast and pelvis/abdomen groups initially only consisted of eight patients who received 155 and 180 fractions, respectively. These concerns were assuaged considerably given the results of the trial period where the datasets for the breast, pelvis, and abdomen groups were roughly double in size and much was learned regarding the approach to handling these sites. The lessons being that tolerances generated for the pelvis and abdomen groups were reasonable, resulting in tolerance violation frequencies of 8.1% and 4.9%, respectively, and that this approach to enforcing tolerance tables was not applicable to breast patients given our current setup technique.

These tolerances were created in order to give a helpful launching point for other centers to adapt a similar strategy that is most useful given their current equipment, setup techniques, and patient population. The advantage of this system is that it can be quickly implemented and is flexible if initial tolerance values need to be updated. In our experience, the initial tolerance values gave a reasonable tolerance violation frequency, but Table 5 shows that the implementation of the indexing system further reduced the expected spread of couch values — for example, abdomen lateral tolerances decreased from 4.2 cm to 2.3 cm. So, as the precision of patient setup begins to evolve, the tolerance values can evolve as well.

## V. CONCLUSIONS

In order to address a potential vulnerability for a geometric miss, we developed a system to prevent mistreatments for the majority of our conventionally fractionated patients. By taking a survey of previously treated patients we developed a system of disease site‐specific tolerances for indexed patients that could be applied to the couch vertical, longitude, and latitude parameters. These tolerances were applied to patients under treatment for disease in the lung, H&N, breast, pelvis, and abdomen sites. All of these sites were indexed with preexisting equipment, except that a simple customizable indexing device was constructed for pelvis sites that allowed for repeatable indexing of the patients feet relative to the treatment couch. Using this system, 90% of treatment fractions fell within our tolerance values, alerting the therapists of a potential mistreatment for 10% of the lung, H&N, pelvis, and abdomen treatments. Due to the many degrees of freedom of our breast setup technique, this method did not prove to be successful for a select subpopulation of patients with larger breasts who tended to have a more variable setup. We also found that the method of updating baseline values after image guidance resulted in less frequent overrides compared to that with a constant baseline value obtained on the first day of treatment. Our system will add an additional layer of safety that could be applied at most clinics with minimal impact on clinical productivity and without the need for additional costly hardware.
